# Selective methioninase-induced trap of cancer cells in S/G_2_ phase visualized by FUCCI imaging confers chemosensitivity

**DOI:** 10.18632/oncotarget.2369

**Published:** 2014-08-19

**Authors:** Shuya Yano, Shukuan Li, Qinghong Han, Yuying Tan, Michael Bouvet, Toshiyoshi Fujiwara, Robert M. Hoffman

**Affiliations:** ^1^ AntiCancer, Inc, San Diego, CA; ^2^ Department of Surgery, University of California, San Diego, CA; ^3^ Department of Gastroenterological Surgery, Okayama University Graduate School of Medicine, Dentistry and Pharmaceutical Sciences, Okayama, Japan

**Keywords:** cell cycle, FUCCI, imaging, S/G_2_ phase block, recombinant methioninase, rMETase, chemotherapy, HeLa cells, MCF-7 cells

## Abstract

A major impediment to the response of tumors to chemotherapy is that the large majority of cancer cells within a tumor are quiescent in G_0_/G_1_, where cancer cells are resistant to chemotherapy. To attempt to solve this problem of quiescent cells in a tumor, cancer cells were treated with recombinant methioninase (rMETase), which selectively traps cancer cells in S/G_2_. The cell cycle phase of the cancer cells was visualized with the fluorescence ubiquitination cell cycle indicator (FUCCI). At the time of rMETase-induced S/G_2_-phase blockage, identified by the cancer cells' green fluorescence by FUCCI imaging, the cancer cells were administered S/G_2_-dependent chemotherapy drugs, which interact with DNA or block DNA synthesis such as doxorubicin, cisplatin, or 5-fluorouracil. Treatment of cancer cells with drugs only, without rMETase-induced S/G_2_ phase blockage, led to the majority of the cancer-cell population being blocked in G_0_/G_1_ phase, identified by the cancer cells becoming red fluorescent in the FUCCI system. The G_0_/G_1_ blocked cells were resistant to the chemotherapy. In contrast, trapping of cancer cells in S/G_2_ phase by rMETase treatment followed by FUCCI-imaging-guided chemotherapy was highly effective in killing the cancer cells.

## INTRODUCTION

### The problem of quiescent cancer cells within a tumor

The resistance of most solid tumors and metastases is a major problem in chemotherapy. The phase of the cell cycle determines to a great extent whether a cancer cell can respond to a given drug. We previously monitored real-time cell cycle dynamics of cancer cells throughout a live tumor intravitally using a fluorescence ubiquitination cell cycle indicator (FUCCI). Approximately 90% of cancer cells in the center and 80% of total cells of an established tumor are in G_0_/G_1_ phase. Similarly, approximately 75% of cancer cells far from (> 100 μm) tumor blood vessels of an established tumor are in G_0_/G_1_. FUCCI imaging demonstrated that cytotoxic agents killed only proliferating cancer cells at the surface, or near blood vessels, and had little effect on the majority of quiescent cancer cells within a tumor. Resistant quiescent cancer cells restarted cycling after the cessation of chemotherapy. Thus, the low chemo-sensitivity of most solid tumors is at least in part due to the large majority of cancer cells in solid tumors being quiescent [1].

FUCCI imaging was used for real-time visualization of the cell cycle kinetics of invading cancer cells in 3-dimensional (3D) Gelfoam® histoculture. Cancer cells in G_0_/G_1_ phase in Gelfoam® histoculture migrated more rapidly and further than the cancer cells in S/G_2_/M phase. After entry into S/G_2_/M phases, cancer cells ceased migrating and restarted migrating after division when the cells re-entered G_0_/G_1_. Migrating cancer cells were resistant to cytotoxic chemotherapy, since they were mostly in G_0_/G_1_ [2].

One solution to the problem of large numbers of cells in G_0_/G_1_ in a tumor is to block the cancer cells in S/G_2_ rather than G_0_/G_1_.

### Methionine dependence

Methionine dependence, the elevated methionine requirement for cancer cell proliferation, is the property of the majority of cancer cell types [3]. There have been several therapeutic strategies to target the methionine dependence of cancer cells. Methionine-starvation therapy, such as with a methionine-free diet or methionine-depleted total parenteral nutrition, prolonged the survival time of tumor-bearing rodents [4, 5]. Methionine-free total parenteral nutrition in combination with chemotherapeutic drugs extended the survival of patients with high-stage gastric cancer [4]. Prostate-cancer patients have been treated with a methionine-depleted diet [5].

A reversible growth arrest of cancer cells has been produced by replacement of methionine in the growth medium by its immediate metabolic precursor, homocysteine, This growth arrest is accompanied by a reduction in the percentage of mitotic cells in the cell population. Furthermore, fluorescence-activated cell sorting demonstrated that the cells are arrested at the S/G_2_ phase of the cell cycle. This is in contrast to a G_1_-phase accumulation of cancer cells, which occurs only in methionine-supplemented medium at very high densities and which is similar to the G_1_ block seen in cultures of normal fibroblasts at high density [6]. The molecular mechanism of the S/G_2_ block has subsequently been investigated [7].

The S/G_2_ trap that that cancer cells enter upon methionine starvation was exploited for selective chemotherapy *in vitro*. In cultures that were initiated with equal amounts of cancer cells and human diploid fibroblasts, substitution of homocysteine and doxorubicin for methionine in the culture medium followed by methionine repletion with vincristine was totally effective at selectively eliminating a methionine-dependent human sarcoma and three methionine-dependent human carcinomas. This chemotherapeutic procedure used was not toxic to normal cells growing alongside the cancer cells and was ineffective when conducted totally in methionine-containing medium [8].

In the present report, we demonstrate that using recombinant methioninase (rMETase) to deplete methionine and thereby trap cancer cells in S/G_2_, and FUCCI imaging to detect the onset of the block, chemotherapy could become effective on the S/G_2_-trapped cancer cells.

## RESULTS AND DISCUSSION

### Recombinant methionine (rMETase) block of cancer cells in S/G_2_ visualized by FUCCI imaging

**Figure 1:**
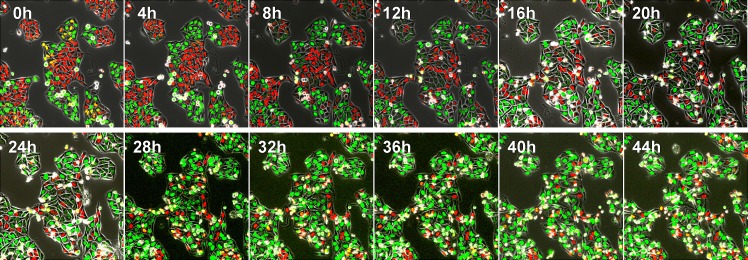
Time-lapse imaging of FUCCI-expressing HeLa cells treated with rMETase After seeding on 35 mm glass dishes and culture over night, HeLa cells were treated with rMETase at a dose of 1.0 unit/ml. All images were acquired with the FV1000 confocal microscope (Olympus, Tokyo, Japan). The cells in G_0_/G_1_, S, or G_2_/M phases appear red, yellow, or green, respectively. Scale bar: 50 μm.

After seeding on 35 mm glass dishes and culture over night, HeLa cells were treated with rMETase at a dose of 1.0 unit/ml. rMETase blocked HeLa and MCF-7 cells in the S/G_2_ phase of the cell cycle as visualized by FUCCI imaging (Figure 1).

**Figure 2:**
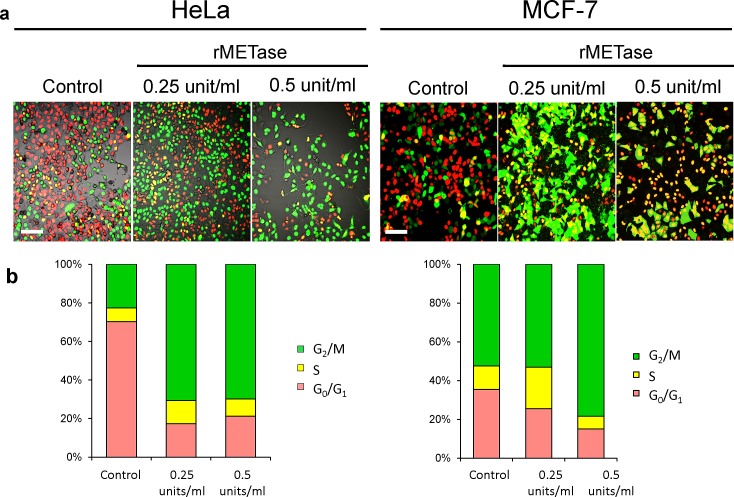
rMETase traps cancer cells in S/G_2_ phase After seeding on 35 mm glass dishes and culture over night, HeLa cells and MCF-7 cells were treated with rMETase, at the indicated doses, for 48 hours. a. Representative images of control or rMETase-treated cells. b. Histogram shows the percentages of cells in G_1_ (red), early S (yellow), or late S/G_2_/M (green). Cells at each cell cycle phase were quantitatively assessed by counting the number of cells with each color. N=5 experiments were analyzed. Scale bars: 50 μm.

After seeding on 35 mm glass dishes and culture over night, HeLa and MCF-7 cells were treated with rMETase either at 0.25 or 0.5 units/ml for 48 hours. FUCCI imaging showed that by 24 hours there was a large shift in the cancer-cell population from G_0_/G_1_ to S/G_2_/M (Figure 2). For HeLa cells, the percentage of cells in G_0_/G_1_ decreased from approximately 80% to approximately 20% by 48 hours in the presence of either 0.25 or 0.5 units/ml rMETase. Approximately 80% of the population became blocked in S/G_2_. For MCF-7 cells, approximately 40% of the untreated cells were in G_0_/G_1_. After 48 hours treatment with 0.25 units/ml rMETase, the percentage of cells in G_0_/G_1_ fell to 20% and with 0.5 units rMETase, the percentage decreased to approximately 15%. Approximately 85% of the cells became trapped in S/G_2_ (Figure 2).

### Cytotoxic chemotherapy drugs killed cancer cells trapped in S/G_2_ by rMETase

**Figure 3:**
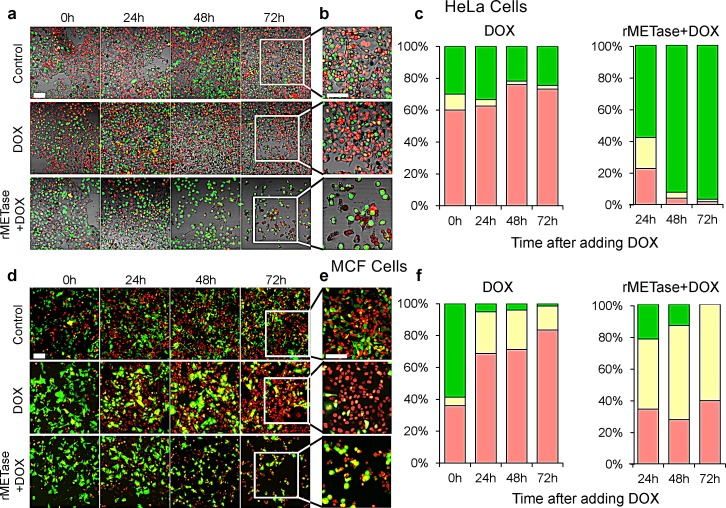
FUCCI cell cycle analysis during chemotherapy with and without rMETase After overnight culture, HeLa cells (a, b and c) and MCF-7 cells (d, e and f) were treated with 0.25 unit/ml rMETase for 48 hours and, then were treated with 0.5 μg/ml doxorubicin (HeLa cells) or 2.5 μg/ml doxorubicin (MCF-7) for 72 hours. For conventional chemotherapy, after culture for 48 hours, HeLa cells (a, b and c) and MCF-7 cells (d, e and f) were treated with 0.5 μg/ml DOX (HeLa cells) or 2.5 μg/ml DOX (MCF-7) for 72 hours. a, b, d, e Representative images acquired with the FV-1000 are shown. c, f, Histograms show the percentages of cells G_1_(red), early-S (yellow), or late-S/G_2_/M (green). Cells at each cell cycle phase were quantitatively assessed by counting the number of cells with each color. N=5 experiments were analyzed. Scale bars: 50 μm.

During rMETase-induced blockage in S/G_2_, doxorubicin (DOX) effectively killed the cancer cells. After overnight culture, HeLa cells and MCF-7 cells were treated with 0.25 unit/ml rMETase for 48 hours. The cancer cells were then treated with 0.5 μg/ml DOX (HeLa cells) or 2.5 μg/ml DOX (MCF-7) for 72 hours. HeLa cells were also treated with 0.5 μg/ml DOX, and MCF-7 cells were treated with 2.5 μg/ml DOX for 72 hours, both without rMETase. With HeLa cells, DOX treatment alone resulted in an increase of cells in G_0_/G_1_ from approximately 60% to 80%. With combination treatment of DOX and rMETase, the number of HeLa cells in G_0_/G_1_ was reduced to approximately 0 with almost all cells blocked in S/G_2_. For MCF-7 cells, approximately 40% of the untreated cells were in G_0_/G_1_ and increased to more than 80% after treatment with DOX alone for 72 hours. In the presence of rMETase and DOX for 72 hours, approximately 40% of the cells were in G_0_/G_1_ (Figure 3).

**Figure 4:**
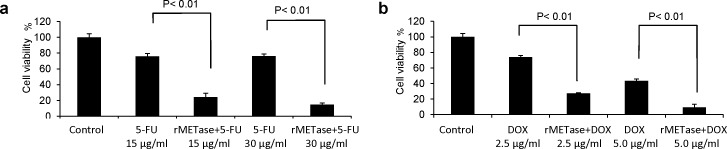
Chemotherapy of S/G_2_-trapped cancer cells Efficacy of combination therapy of rMETase and 5-FU (a); rMETase and DOX (b); on FUCCI-expressing MCF-7 cells. Cell viability was assessed by counting living cells compared to control. Data bars means ± SD of triplicate samples.

DOX alone, at 2.5 μg/ml, killed approximately 25% of the MCF-7 cells. The combination of DOX, at 2.5 μg/ml, and rMETase, at 0.25 units/ml, killed approximately 80% of the cells (*P*<0.01 compared to DOX alone). DOX, at 5 μ/ml, and rMETase, at 0.25 units/ml, killed approximately 90% of the cells (*P*<0.01 compared to DOX alone) (Figure 4).

For MCF-7 cells treated with 5-FU, at 15 μg/ml, approximately 40% of the cells were killed. With the combination of rMETase (0.25 units/ml) and 15 μg/ml 5-FU, approximately 80% of the cells were killed (*P*<0.01 compared to 5-FU alone). With 5-FU, at 30 μg/ml, approximately 40% of the cells were killed, and the combination of 5-FU, at 30 μg/ml, and 0.25 units/ml rMETase, approximately 90% of the cells were killed (*P*<0.01 compared to 5-FU alone) (Figure 4).

The strategy and technology described in this report, whereby cancer cells are selectively and synchronously trapped by rMETase in S/G_2_, the most drug-sensitive phase of the cell cycle, where they were identified by FUCCI imaging and then treated with S/G_2_-phase-specific-drugs was highly effective compared to standard chemotherapy.

Previously developed concepts and strategies of highly selective tumor-targeting [9-20] can take advantage of spatial–temporal cell-cycle imaging of cancer cells described in the present and previous [1, 2, 21] reports.

Previously, different methods of cancer-cell synchronization have been used in order to sensitize the cells to chemotherapy. Such methods include chronotherapy which attempts to target the time of day when most cancer cells in tumors are thought to be dividing [22]. Excess thymidine or its analogs have also been used to arrest cancer cells in S-phase, where they are sensitized to S-phase drugs such as 5-FU or methotrexate, and after release of the block, the cancer cells enter M-phase synchronously where they are sensitive to M-Phase drugs such as paclitaxel [23-25].

Cancer-cell synchronization with cell-cycle-phase-specific drugs, such as cytosine arabinoside, methotrexate and hydroxyorea have also been carried out [26-30], for example, to block cells in S-phase which can sensitize the cancer cells to an M-phase drug, such as paclitaxel, administered after the S-phase block is lifted [26-30].

The calcium channel blocker mibefradil has been used to synchronize glioblastoma cells at the G_1_/S checkpoint, thereby making the glioblastoma cells sensitive to first-line therapy temozolomide [31]. Statins, such as Lovastatin, can be used to synchronize cancer in G_1_ by preventing formation of an early intermediate in the cholesterol pathway essential for progression of cells through early G_1_ phase [32, 33]. After the block is lifted, the cancer cells can be effectively treated with an S-phase drug.

PDO332991, a pyridopyrimidine, has been shown to be a selective inhibitor of cyclin-dependent kinases 4 and 6 and induced early-G_1_ arrest in primary human myeloma cells and other cancer cell types, including breast cancer *in vitro* and in cancer xenograft models. As PDO0332991 acts reversibly, it can be used as a synchronizing agent and when used for sequence combination with cytotoxic agents is active against myeloma cells *in vitro* and *in vivo* [34]. A cyclin-dependent kinase inhibitor RO-3306 reversibly arrests 95% of treated cells in G_2_ phase. These cells rapidly enter mitosis after the block is lifted and become sensitive to M-phase drugs [35]. Growth factors such as EGF, G-CSF, and IL-6 can stimulate cancer cell out of G_0_, making them sensitive to chemotherapy agents such as docetaxel [36-38]. Reviews on cell synchronization are available [39-42].

The critical advantage of rMETase synchronization (blockage) is that, unlike the methods described above, it is cancer specific [3,6,8,43-51].

## CONCLUSIONS

A major problem for successful chemotherapy is the very high percentage of quiescent G_0_/G_1_ cancer cells in a tumor. The present report has demonstrated a solution to the problem by selectively trapping cancer cells in S/G_2_, with recombinant methioninase (rMETase). The S/G_2_-trapped cancer cells became sensitive to chemotherapy which targets cells in this phase of the cell cycle, which are the majority of the most widely-used chemotherapy drugs. Alternatively, the rMETase-induced S/G_2_ block can be lifted and the cells can become sensitive to M-phase drugs. This approach has significant clinical potential since almost all cancer cell types tested are methionine dependent and arrest in S/G_2_ when deprived of methionine with an agent such as rMETase.

## MATERIALS AND METHODS

### Recombinant Methioninase (rMETase)

Recombinant L-methionine α-deamino-γ-mercaptomethane lyase (methioninase, METase) [EC 4.4.1.11] from *Pseudomonas putida* has been previously cloned and was produced in *Escherichia coli* (AntiCancer, Inc., San Diego, CA). rMETase is a homotetrameric PLP enzyme of 172-kDa molecular mass [52].

### FUCCI (Fluorescence ubiquitination cell cycle indicator)

The FUCCI probe was generated by fusing mKO2 (monomeric Kusabira Orange2) and mAG (monomeric Azami Green) to the ubiquitination domains of human Cdt1 and geminin, respectively. These two chimeric proteins, mKO2-hCdt1(30/120) and mAG-hGem(1/110), accumulate reciprocally in the nuclei of transfected cells during the cell cycle, labeling the nuclei of G_1_ phase cells red and nuclei of cells in S/G_2_ phase green [53].

### FUCCI-expressing HeLa cells and MCF-7 cells

Plasmids expressing mKO2-hCdt1 or mAG-hGem (MBL, Nagoya, Japan) were transfected into HeLa cells and MCF-7 cells. HeLa cells were grown in DMEM supplemented with 10% fetal bovine serum and penicillin/streptomycin. MCF-7 were grown in MEM-supplemented with L-glutamine and 10% fetal bovine serum and penicillin/streptomycin [53].

### Imaging of FUCCI-expressing cancer cells

Time-lapse images of HeLa and MCF-7 cells stably transfected with FUCCI vectors were acquired using a confocal laser scanning microscope (FV1000; Olympus, Tokyo, Japan) [1, 2, 21].

### Cell viability

For cell viability determinations before and after chemotherapy, with and without rMETase, the cells were stained with crystal violet, and the relative number of cells was quantified using ImageJ (NIH, Bethesda, MD).

### DEDICATION

This paper is dedicated to the memory of A. R. Moossa, MD.

## Abbreviations

rMETase: recombinant methioninase
FUCCI: fluorescence ubiquitination cell cycle indicator
